# Estimation of HIV-Testing Rates to Maximize Early Diagnosis-Derived Benefits at the Individual and Population Level

**DOI:** 10.1371/journal.pone.0053193

**Published:** 2013-01-07

**Authors:** Dario A. Dilernia, Daniela C. Monaco, Carina Cesar, Alejandro J. Krolewiecki, Samuel R. Friedman, Pedro Cahn, Horacio Salomon

**Affiliations:** 1 Centro Nacional de Referencia para el SIDA, Facultad de Medicina, Universidad de Buenos Aires, Ciudad Autonoma de Buenos Aires, Argentina; 2 Investigaciones Clinicas, Fundación Huésped, Ciudad Autonoma de Buenos Aires, Argentina; 3 National Development and Research Institutes, Inc., New York, New York, United States of America; University of Ottawa, Canada

## Abstract

**Background:**

In HIV infection, initiation of treatment is associated with improved clinical outcom and reduced rate of sexual transmission. However, difficulty in detecting infection in early stages impairs those benefits. We determined the minimum testing rate that maximizes benefits derived from early diagnosis.

**Methods:**

We developed a mathematical model of HIV infection, diagnosis and treatment that allows studying both diagnosed and undiagnosed populations, as well as determining the impact of modifying time to diagnosis and testing rates. The model’s external consistency was assessed by estimating time to AIDS and death in absence of treatment as well as by estimating age-dependent mortality rates during treatment, and comparing them with data previously reported from CASCADE and DHCS cohorts.

**Results:**

In our model, life expectancy of patients diagnosed before 8 years post infection is the same as HIV-negative population. After this time point, age at death is significantly dependent on diagnosis delay but initiation of treatment increases life expectancy to similar levels as HIV-negative population. Early mortality during HAART is dependent on treatment CD4 threshold until 6 years post infection and becomes dependent on diagnosis delay after 6 years post infection. By modifying testing rates, we estimate that an annual testing rate of 20% leads to diagnosis of 90% of infected individuals within the first 8.2 years of infection and that current testing rate in middle-high income settings stands close to 10%. In addition, many differences between low-income and middle-high incomes can be predicted by solely modifying the diagnosis delay.

**Conclusions:**

To increase testing rate of undiagnosed HIV population by two-fold in middle-high income settings will minimize early mortality during initiation of treatment and global mortality rate as well as maximize life expectancy. Our results highlight the impact of achieving early diagnosis and the importance of strongly work on improving HIV testing rates.

## Introduction

In the last twenty years, development of new antiretroviral drugs for HIV infection has improved the effectiveness of treatment. This has led to a delay in AIDS progression, a reduction in mortality rates, and a partial restoration of the immune system in infected individuals through normalization of the CD4-count [1], [2], [3], [4]. Moreover, clinical trials have shown that early initiation of antiretroviral therapy reduces rates of sexual transmission [5] supporting expansion of treatment as a strategy for prevention of new infection [6], [7], [8].

However, difficulty in detecting HIV infection in early clinical stages significantly impairs treatment-derived benefits [9], [10], with late detection being associated with higher risk of morbidity and mortality, reduced effectiveness of treatment and increased risk of transmission [11], [12], [13], [14], [15], [16], [17]. In spite of consistent efforts to increase accessibility to antiretroviral drugs, less than half of HIV infected individuals actually have access to them. In low- and middle-income settings an average treatment coverage of 31% (27%–34%) was reported [18]. Moreover, the majority of individuals infected with HIV are unaware of their serological status [18] and previous studies have reported a prevalence of late presenters of 30–40% in developed settings [9], [10], [16].

Inability to detect HIV infection in early stages is mainly associated with the long asymptomatic stage of the natural infection together with additional factors like socio-economic status, social stigma, under-perception of risk and the effectiveness of testing strategies to overcome those barriers. Furthermore, because of these limitations there is a significant uncertainty regarding clinical and epidemiological features of the hidden undetected population living with HIV which in turn leads to uncertainty about the global HIV epidemic [18].

In the present study we develop a Markov model of infection, diagnosis and treatment that allows predicting clinical status of patients at different stages of infection and treatment as well as estimating global mortality rates and life expectancy for people living with HIV. By running the model under different initial conditions we determine the impact that reductions in the time to diagnosis, different algorithms for initiating treatment and different efficiencies of detecting HIV infection can have on infection outcomes. Finally, we determine the HIV detection rates that maximize treatment-derived benefits.

## Materials and Methods

### Model Design

**Figure 1:**
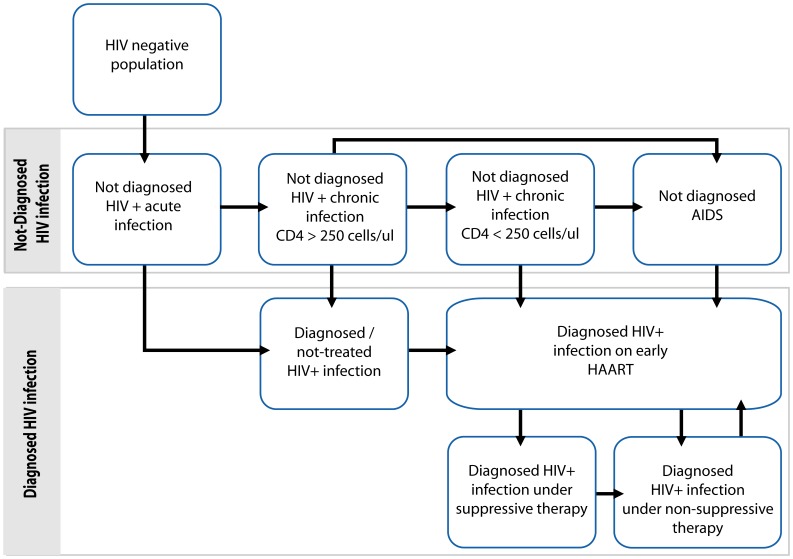
Schematic view of the model structure. This flowchart shows the states that constitute the model, in which individuals can be classified at any time of the simulation. For additional details on probabilities and logical rules involved in transition between states see supplemental material.

We used a Markov model built in TreeAge Pro Suite v.1.0.2. Sources of data are detailed in table 1 and explained in more detail in Text S1. The model is composed of 9 mutually exclusive states represented in Figure 1: the state (i) “*HIV negative population*” contains all the uninfected individuals, the states (ii) “*Not diagnosed - Acute Infection*”, (iii) “*Not diagnosed – CD4>250*”, (iv) “*Not diagnosed – CD4<250*” and (v) “*Not diagnosed – AIDS*” represent the HIV positive undiagnosed individuals at different stages of infection. The following states group diagnosed individuals according to treatment stage: the state (vi) “*Diagnosed – Not treated HIV infection*” includes all the diagnosed but yet untreated HIV positive individuals, the state (vii) “*Diagnosed infection on early HAART*” includes all the diagnosed patients in the first 6-months of therapy under any of the three available HAART regimens, the state (viii) “*Diagnosed infection under suppressive therapy*” includes all the diagnosed patients successfully treated (maintaining viral load under 400 copies/ml) and the state (ix) “*Diagnosed infection under non-suppressive therapy*” includes all diagnosed patients under non-suppressive therapy (because of treatment failure before scheduled viral load or because of unavailability of a new HAART regimen after several treatment failures).

**Table 1:** Detail of the INPUTS for the model.

			Expectedvalue	Range		Ref.
***Constant Variables***						
	Patient´s age at the moment of infection (year-old)		24	(18 to 56)		[19]
***Tracker Variables***						
	Initial CD4-count (CD3+CD4+ cells/µl)		800	(600 to 1000)		Assumed
		CD4 change during viral suppression (cells/µl*year)	60	(40 to 80)		[3]
		CD4 change after dual response (cells/µl*6 first months of therapy)	148	(102 to 225)		[22]
		CD4 change after immunologic-only response (cells/µl*6 first months of therapy)	125	(85 to 194)		[22]
		CD4 change after virologic-only response (cells/µl*6 first months of therapy)	0	0		[22]
		CD4 in non-responders (cells/µl*6 first months of therapy)	0	0		[22]
	Initial Viral-load (VL; log_10_[RNA copies/ml])		0			
		VL when become infected (log_10_ [RNA copies/ml])	4.6	(3.6 to 5.6)		[23], [24], [32], [56]
		VL at rebound after therapy failure (log_10_ [RNA copies/ml])	4.1	(3.6 to 4.6)		[25], [26]
		VL change after dual response (log_10_RNAcopies/ml*6 first months of therapy)	−3.1	(−3.6 to −2.5)		[22]
		VL change after immunologic-only response (log_10_RNAcopies/ml*6 first months of therapy)	−1.6	(−2.2 to −0.8)		[22]
		VL change after virologic-only response (log_10_RNAcopies/ml*6 first months of therapy)	−2.9	(−3.4 to −2.2)		[22]
		VL change in non responders (log_10_RNAcopies/ml*6 first months of therapy)	−0.7	(−1.7 to 0)		[22]
***Functions***						
	VL-dependent decay of CD4-count					[20]
	Probability of AIDS progression					[27], [29]
	Probability of death from AIDS					[28], [29]
	Probability of detection through symptoms					[30]
***Rates***						
	CD4 decay factor		14.76	(9.39 to 20.43)		[20]
	Toxicity in the first 6 months of treatment (% patients experiencing toxicity/treated patients*year)		28.6	(24 to 33.2)		[21]
	Toxicity after the first 6 months of treatment (% patients experiencing toxicity/treatedpatients*year)		15.4	(13.4 to 17.4)		[21]
	Rebound rate in the first year (% patients failing/successfully treated patients*year)					[21]
		First HAART regimen	3.1	(2 to 4.2)		
		Second HAART regimen	6.2	(4 to 8.4)		
		Third HAART regimen	12.4	(8 to 16.8)		
	Rebound rate after the first year (% patients failing/successfully treated patients*year)					[21]
		First HAART regimen	5·2	(4.6 to 5.9)		
		Second HAART regimen	10·4	(9.2 to 11.8)		
		Third HAART regimen	20·8	(18.4 to 23.6)		
	Suppression rate (% patients achieving viral load bellow 400 RNA copies/ml*treated patients*6first months of therapy)					[22], [23], [24]
		First HAART regimen	75	(65 to 85)		
		Second HAART regimen	65	(55 to 75)		
		Third HAART regimen	30	(20 to 40)		
	Rate of dual response to treatment after suppression (VR+ IR+, % patients achieving undetectableviral load (<50 RNA copies/ml)/patients successfully suppressing viral load (<400 RNA copies/ml))		67	(50 to 90)		[22]
	Rate of discordant responses					[22]
		Immunological not virological response (% patients improving CD4 count/patients notsuccessfully suppressing viral load)	60	(40 to 80)		
		Virological not immunological response (% patients reducing viral load between 50–400 RNA copies/ml)	33	(10 to 50)		
	Risk associated to past AIDS (folds of increased risk)		2.35	(1.48 to 3.71)	[21]	
***Other parameters***						
	Time allowed to maintain the same regimen in spite of not virologic response		6 months			Assumed
	Frequency of viral-load testing		6 months			Assumed
	Number of available HAART regimens		3			Assumed

### Analytical Approach

The Markov model is run in a sequence of cycles. In the present model each cycle represents a time step of one month and each simulation lasts 1,800 cycles in order to allow the simulation to converge. At each cycle, transitions between the 9 mutually exclusive states described above are determined by rates, logical rules, and probabilities dependent on individual-level parameters. In this sense, individual-level simulations were performed by applying a Monte-Carlo method of analysis. This not-deterministic method provides more flexibility compared to standard Markov cohort analyses since by using tracker variables it retains memory of previous events from one cycle to the next. A total of 10,000 simulations were run per analysis.

### Initial Conditions for the Model

Individuals enter the model homogeneously distributed across the 1,800 cycles of simulation. The “entering cycle” is recorded as the moment of infection and defines the initiation of the simulation of a “patient’s life”. At that cycle, each patient is linked to an initial value of CD4 count (800±200 cells/µl), viral load (4.6±1.0 Log_10_[copies/ml]) and age (18 to 56, [19]) sampled from observed distributions of CD4 count in healthy patients, viral load at the chronic stage of natural infection and age at the moment of infection, respectively (see Text S1).

### Model Evolution During the Time from Infection to Diagnosis

During the cycles between the moment of infection and the moment of diagnosis, the viral load remains constant and the CD4 count diminishes at a rate dependent on the viral load, following a function adjusted to data published by Mellors *et al* [20] (Figure S1). When CD4 count drops below the level of 250 cells/µl a probability of AIDS progression dependent on the patient´s age at the moment of infection, CD4 count, viral load and patient´s current age, is applied at each cycle. After progressing to AIDS, a probability of death associated with HIV infection is applied as well as a probability of being diagnosed by symptomatology. The last is further described at follows. A background probability of dying due to other causes based on general population age-dependent mortality rates is applied at every cycle.

### Modeling HIV Diagnosis

In the present model, HIV positive individuals may achieve diagnosis through three different routes: (i) in this deterministic route, individuals are diagnosed when they reach a cycle on the simulation whose value equals the entering cycle (moment of infection) plus a pre-defined fixed value (time from infection to diagnosis), this is, individuals are diagnosed a fixed time after infection that is the same for every simulation of the same run. (ii) In this probabilistic route, individuals are diagnosed through symptomatology. This route is only feasible after an undiagnosed individual has progressed to AIDS and the probability is defined by a function dependent on the current CD4 count and pre-defined for a maximum annual detection rate for patients with CD4 count lower than 50 cells/µl (see supplemental material). (iii) In this probabilistic route, HIV diagnosis occurs as a consequence of applying an annual testing rate over the total undiagnosed HIV positive population. Combinations of these three routes define the analytical strategies described as follows.

### Analytical Strategies

Simulations were run using two different strategies: In the first strategy, in order to analyze benefits from earlier diagnoses of HIV infection, diagnosis routes (i) and (ii) described above were applied, diagnosis route (iii) was excluded, and the pre-defined fixed value for “time from infection to diagnosis” was set in each run at a different value in a range from 2 years to 16 years with 2-year intervals. In addition, in order to take into account the variability of outcomes associated with initiating HAART at different CD4 count and detecting AIDS cases through symptomatology with different efficiencies, each run performed at each fixed “time from infection to diagnosis” was repeated under each of the 6 combinations of the following situations: Initiating HAART at CD4 count equal or lower than 200 cells/µl, 350 cells/µl or 500 cells/µl; and diagnosing AIDS through symptomatology with an efficiency that allows annual detection of 35% or 75% of patients with AIDS. In a second analytical strategy aimed at understanding the impact of modifying the annual detection rate, diagnosis route (i) was excluded (i.e. time from infection to diagnosis was allowed to vary free) and diagnosis routes (ii) and (iii) were applied. Then an annual testing rate, defined as the proportion of undiagnosed individuals that are diagnosed per year was applied to all the undiagnosed HIV-positive individuals, independently of their CD4 count, and modified in a range between 0.1 and 50%.

### Modeling HIV Treatment

When the diagnosis is achieved, patients remain diagnosed and untreated until the condition to initiate HAART is satisfied (i.e. CD4 count lower than a specified value). After initiating HAART, transitions will be limited to three final stages containing patients initiating a new or first HAART regimen, patients under successful therapy and patients under non-suppressive therapy. During this last phase of the simulation, rates and probabilities obtained from literature and describing toxicity of HAART regimens [21], success in achieving undetectable viral load [22], [23], [24], discordant responses to treatment [22] and viral rebound [21] are included. Also, viral load and CD4 count are modified according to the different treatment outcomes and in a magnitude that resembles that previously reported in the literature [2], [22], [25], [26]. In the present model the number of available HAART regimens is limited to three, after which the patient is retained in the stage representing non-suppressive therapy, while viral load is tested on a 6-month basis frequency. At each cycle of the simulation, CD4-count and viral load-dependent probabilities of progression to AIDS and death related to AIDS are applied. A more detailed description of transition between states can be found in Text S1.

### Modeling Disease Progression and Death Rates

The function determining the probability of progression to AIDS is based in the one previously described by Phillips *et al* [27], modified in order to account for patient’s age at the moment of infection. The probability of progression to AIDS is applied at every cycle of the simulation. It is not defined *a priori* but is, instead, determined by the CD4 count, the viral load and the patient’s age at each cycle of the simulation. In the model, individuals can die due to causes related to AIDS and due to causes not related to AIDS. The former depends on patient’s age at infection and is only applied after a patient has progressed to AIDS and until the AIDS condition is resolved. The death rates not related to AIDS are applied at every cycle of the simulation, and are derived from age-dependent mortality rates of general population. A detailed description of these functions can be found in Text S1 and in Figure S2.

### Assessment of Internal Consistency

In order to ensure the mathematical consistence of the model a lower limit for viral load was set at 1.7 Log_10_[copies/ml] (which corresponds to undetectable viral load with a sensitivity of 50 copies/ml), and at 50 cells/µl for CD4 count; as well as an upper limit for viral load was set at 5.6 Log_10_[copies/ml], while for CD4 count it was set at the value initially sampled for the CD4 count previous to infection. These limitations prevent inconsistent prediction of variations of CD4 count and viral load while unlimited reduction in CD4 count or extreme decay rates are prevented by using a function adjusted to data published by Mellors *et al* [20] (see Text S1 for function development and Figure S1). As mentioned above, the function describing the probability of progression to AIDS and the probability of death after AIDS was created based on previously described functions [27], [28] and adjusted with data previously published [29] for taking into account patient´s age at the moment of infection. The function describing the probability of achieving the diagnosis through symptomatology was defined as a linear function dependent on CD4 count, based on the one previously described by Sanders *et al* [30], but modified to allow a maximum rate of detection of 35% or 75% (DTS efficiency) at CD4 count equal or lower to 50 cells/µl and a minimum of 0% at CD4 count equal or higher than 350 cells/µl.

### Assessment of External Consistency

In order to assess the external consistency, the model was run under two different situations. In the first one, the possibility of getting diagnosed was removed and the model was set to sample individuals’ age at infection from a uniform distribution in order to have equal numbers of individuals at different age ranges. Then, the time to AIDS and death were determined by subtracting the individuaĺs age at infection from the individuaĺs age at disease progression or the individuaĺs age at death, respectively. Predictions were compared with those previously reported for CASCADE Cohort [29] (see Text S1 and Figures S6, S7, S8 and S9 for further details). For the second situation, and in order to assess the external consistency of our model during simulations involving diagnosis and treatment, populatiońs diagnosis rate was set at 10% (per 100 undiagnosed HIV-positive persons per year) and a Kaplan Meier analysis was performed, censoring cases at the moment of death due to other causes and considering death related to AIDS as the event. In these simulations, individualś age at infection were sampled from the distribution previously reported [19] and individuals who died before achieving diagnosis or individuals still alive at the end of the simulation were excluded from the analysis. The function describing age-specific mortality rates was obtained by deriving the natural logarithm transformation of the survival curve and the average mortality rate per age range was estimated by integrating the function between each analytical range (see Text S1 and Figures S10, S11, S12 and S13 for further details).

### Statistical Considerations

The model was run with 10,000 Monte-Carlo simulations of 1,800 cycles per simulation. The majority of outputs were described by proportions, in which case the 95% confidence intervals were estimated assuming normal distribution due to the large number of cases. Confidence intervals for proportion were also estimated by bootstrap techniques demonstrating identical results. In all the analyses performed, calculations demonstrated a power higher than 99% to detect significant differences with an alpha level of 0.05. For life-expectancy analysis, one-way ANOVA was performed; homogeneity of variances was assessed by Levenés test and *a-priori* defined *post-hoc* multiple comparisons were assessed by Bonferronís test.

## Results

**Figure 2:**
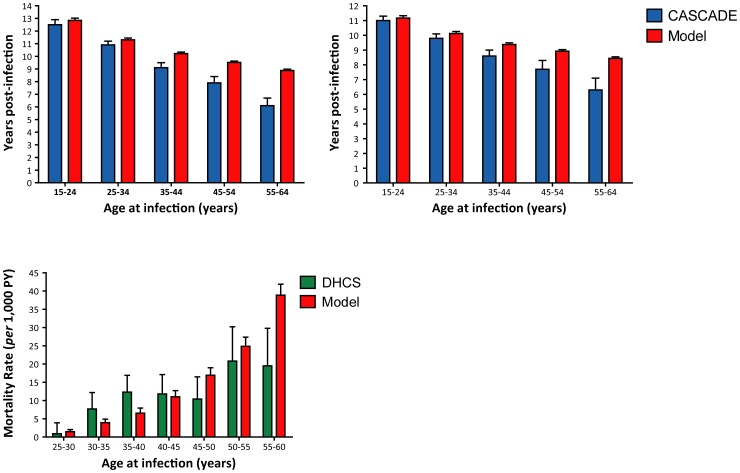
Comparison of modeĺs outputs related to mortality rates with those previously reported. Time to AIDS and time to death as well as age-dependent AIDS-related mortality rates are compared with those previously reported for the CASCADE cohort and the DHCS cohort.

In order to validate the model, predictions regarding time to AIDS and death during the natural history of infection as well as the mortality rate during treatment were compared with data previously published. As shown in Figure 2, modeĺs predictions are similar to those previously reported for patients without treatment or under monotherapy, being more accurate for individuals getting infected before 45-year old. In order to assess the external consistency of the model during simulations involving diagnoses and treatment, diagnosis rate was set at 10% (per 100 undiagnosed HIV-positive persons per year). This testing rate was used because is the one that most closely resembles observations from high-income settings as explained later. Then, age-specific survival rates were obtained from the survival curve for AIDS-related death in the diagnosed population. As shown in Figure 2, model ´s predictions of mortality rate after diagnosis are consistent with those previously observed.

**Figure 3:**
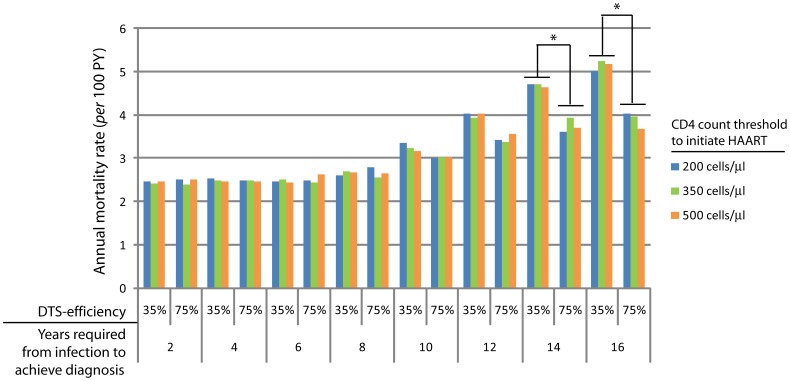
Annual mortality rate of total individuals living with HIV for each of the analytical settings. The model was run with the different fixed times from infection to diagnosis detailed in the figure. At the end of each run, the annual mortality rate was determined. Each run was repeated with the 6 combinations of the three CD4 count threshold to initiate HAART (200, 350 and 500 cells/µl) and two different efficiencies to detect HIV infection through symptomatology (35% and 75%). *Significantly different with a *p*-value<0.05.

Next, we analyzed the impact of modifying the diagnosis delays in mortality rate of the HIV-infected population, including in the analysis both diagnosed and undiagnosed individuals. For this analysis, time from infection to diagnosis was set as a fixed factor and the model was run for 35% and 75% of DTS efficiency and for 200, 350 and 500 cells/µl treatment CD4 thresholds. The analyses show that achieving diagnosis during the first eight years of infection, the annual mortality rate stays stable at an average of 2.5 deaths per 100 infected individuals per year (Figure 3). For higher delays, this rate significantly increases in a DTS capacity-dependent manner reaching an average value of 3.8 for an annual DTS-capacity of 75% and 5.0 for an annual DTS-capacity of 35%. These predictions reflect the fact that when diagnosis is achieved in early stages of infection patients are not advanced enough to allow detection-through-symptomatology; while, if diagnosis is achieved at advanced stages, then the capacity to detect infection-through-symptomatology plays an important role in preventing higher rates of mortality. Also, as shown in Figure 3, the CD4-count threshold at which treatment is initiated does not impact significantly on the overall mortality rate at the population-level. Small variations can be observed in the estimations obtained from runs performed with different treatment CD4 thresholds under the same diagnosis delay; however those variations are likely to be related with the probabilistic estimation of the proportion in the model since none statistically significant difference was observed.

**Figure 4:**
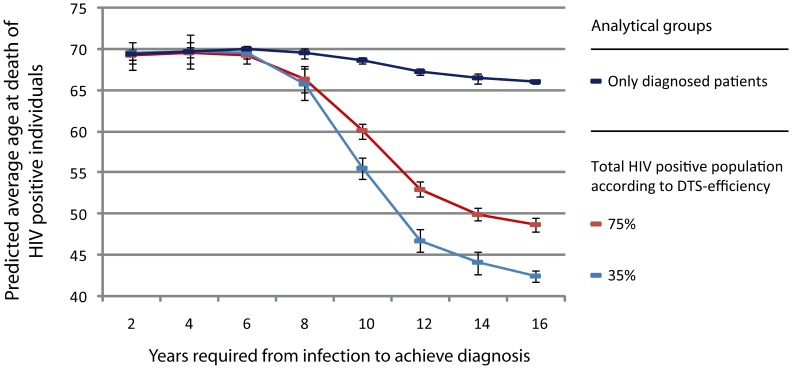
Comparison of average age at death of HIV positive individuals according to the capacity of detecting infection by symptomatology and the access to treatment. Age at death for the population living with HIV are compared with those individuals who have achieved diagnosis. Predictions show the impact of early diagnoses on extending life of individuals living with HIV, as well as show that improvements in efficiency of detecting HIV infections through symptomatology can significantly extend life expectancy in cases where the diagnosis is achieved later than 8 years post-infection. Predictions show the major impact that access to therapy can have on extending life expectancy even for patients diagnosed in advanced stages of infection.

When patientś ages at death obtained from the simulations run above are analyzed, a strong association between life expectancy and diagnosis delay can be observed (Figure 4). According to our results, life expectancy for the total HIV population is increased up to 27 years comparing individuals diagnosed during the first 8 years of infection with the worst scenario analyzed of 16 years-delay with a 35% DTS-capacity. An improvement in the DTS-capacity can significantly increase life expectancy when the diagnosis is achieved at or after 10 years of infection (6.2 years when comparing life expectancy at 16 years-delay for both DTS efficiencies, *p*<10^−4^). When the analysis is limited to diagnosed individuals (i.e. excluding individuals who die before diagnosis), life expectancy variations are lower and less sensitive to diagnosis delay but still significantly different when the diagnosis is achieved during the first 12 years of infection compared to a later diagnosis (Figure 3, *p*<10^−11^). As was the case for annual mortality rate, CD4-count threshold at which treatment is initiated does not impact significantly on the average age at death of patients in our simulations.

**Figure 5:**
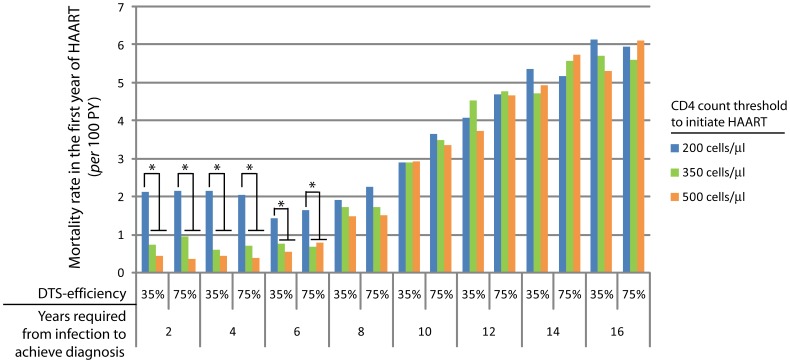
Mortality rate in the first year of HAART for each of the analytical settings. For the present analysis, the percentage of newly treated patients that die during the first year of initiation of the first HAART regimen was estimated. In this case, data recovered across the whole simulation was analyzed by identifying patients that have initiated HAART and whose date of death occurred within the 12 simulation cycles after initiation of HAART, over the total individuals that have initiated HAART during the model run. *Significantly different with a *p*-value<0.05.

**Figure 6:**
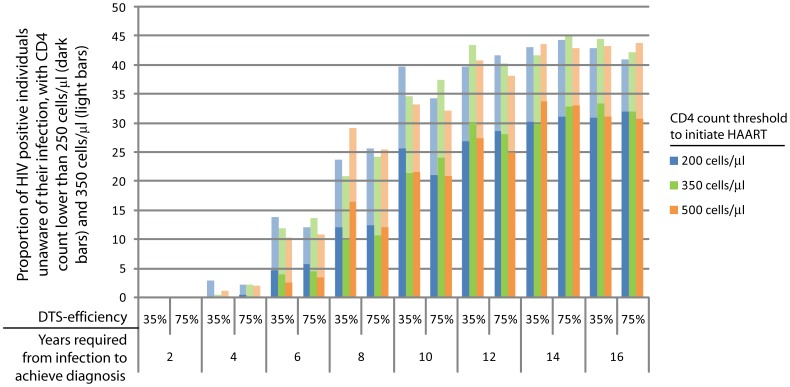
Proportion of HIV positive individuals unaware of their infection status having CD4 counts below 250 (dark bars) or 350 cells/µl (light bars), for each of the analytical settings. In this case, model’s outputs were further analyzed to determine the proportion of HIV positive individuals unaware of their infection whose CD4 count have drop below critical levels (250 and 350 cells/µl).

Based on results shown above, the time point of 8 years of infection seems to be an inflection point: a diagnosis achieved after 8 years of infection leads to significant increases in mortality rate and a decreased age at death while a diagnosis achieved before that time of infection leads to a stable mortality rate and a stable expected age at death, independently of diagnosis delay and suggesting equal benefits derived from diagnoses achieved at any time point during the first 8 years of infection. However, a strong dependence of analyzed mortality rate during the first year of treatment on CD4 count at initiation of HAART was observed: as shown in Figure 5, mortality rates are significantly higher in patients for whom treatment initiation was deferred to 200 cells/µl (2.1% for 2 years-delay) compared with initiation at 350 or 500 cells/µl (0.8% and 0.4% respectively for the same delay, *p*<0.001). In addition, the model predicts that after 8 years-delay, this rate increases abruptly in a linear manner until reaching an average of 5.5 deaths per 100 infected individuals per year at 16 years-delay in a DTS-capacity independent manner. The association of early mortality rate with CD4 count at initiation of HAART is lost for diagnosis delays equal to or higher than 8 years since according to our prediction after that time of infection patients are diagnosed harboring a median CD4 count of 200 cells/µl (Figure S3). In this sense, as the diagnosis delay becomes greater, the proportion of undiagnosed individuals with CD4 counts at critically lower levels become higher. As shown in Figure 6, when diagnosis is achieved later than four years after infection, undiagnosed HIV positive individuals with CD4 counts lower than 250 or 350 cells/µl starts to become an increasing proportion of the total undiagnosed population. Considering our previous results, Figure 6 shows that if diagnosis is not achieved during the first 8 years of infection, 20–25% of the undiagnosed population will have a CD4 count lower than 350 cells/µl and will be therefore excluded from benefits derived from an earlier initiation of treatment while 10–15% will be in urgent need of treatment (i.e. CD4 count lower than 250 cells/µl). Although no differences in the composition of the undiagnosed population can be expected for settings having different treatment CD4 threshold but the same diagnosis delay, predictions derived from runs obtained for all the situations are still shown in Figure 5 as a demonstration of the variability associated to independent runs.

**Figure 7:**
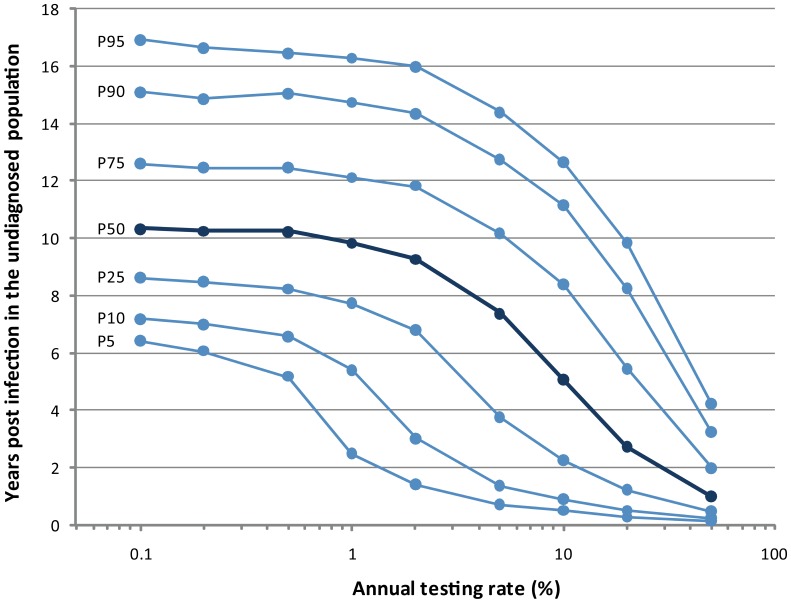
Analysis of the impact of detection rate on diagnosis delay. The data was obtained from simulations where the diagnosis delay was relaxed. Then testing rate was modified and distribution of year of infection in the undiagnosed population was analyzed. In the figure, each curve corresponds to a different percentile of that distribution. In dark blue is shown the curve for the median.

At the population-level, reduced capacity of diagnosing HIV positive individuals in early stages of infection leads to a greater proportion of HIV-positive individuals being unaware of their infection. In our model, diagnosis delay is directly related to the proportion of individuals living with undiagnosed infection: The longer it takes to achieve a diagnosis, the higher the proportion of individuals living with undiagnosed infection (Figure S4). Inversely, to increase and sustain testing frequency would lead to an accumulation of probabilities over time for each individual to get diagnosed and to the consequent reduction in diagnosis delay. In this sense, in order to analyze the relationship between time of infection and testing rate in the undiagnosed population, the time from infection to diagnosis was allowed to vary free while the testing rate was set as a fixed factor. Then, the model was run under different testing rates and the distribution of time of infection predicted for the undiagnosed population was analyzed for each rate. As shown in Figure 7, both variables are related in a non-linear manner. The percentile lines specify the proportion of undiagnosed individuals with less than a specific time of infection for each testing rate. When the modeĺs outputs from the simulations using route 3 (fixed testing rate, relaxed diagnosis delay) are compared with outputs from simulations using route 1 (fixed diagnosis delay) we observe that predictions obtained with a specific annual testing rate correspond to those obtained when diagnosis delay is fixed at the third quartile of the heterogeneous distribution of diagnosis delays observed for that rate. In other words, predictions obtained using a specific fixed diagnosis delay are those expected for a real-world setting were the 75% of the HIV-positive population is diagnosed within those first years of infection (Figure S5).

When the relationship between testing rates and diagnosis delay is analyzed, according to modeĺs predictions a median and stable time to diagnosis between 10 and 11 years is associated with a testing rate lower than 1%. In that situation, diagnosis is achieved exclusively though symptomatology at the mentioned rate. Importantly, according to our predictions, to achieve an annual testing rate of 20% will allow diagnosis of 90% of HIV-positive individuals within the first 8 years of infection, which in turn, and based on our previous predictions, will minimize mortality rate, maximize life expectancy and achieve diagnosis in a clinical stage where early mortality at initiation of treatment can be prevented by treatment strategies based on higher CD4 thresholds.

## Discussion

Based on the known impact that treatment has in quality and expectancy of life of HIV positive individuals, to reach an early diagnosis and to ensure access to therapy stands as one of the major goals in the fight against HIV epidemic [31]. While it is clearly known how strongly dependent on diagnosis delay are the clinical status of patients at diagnosis and the mortality rates [9], [10], [16], [17], our model provides a deeper description about the way they are interrelated.

**Figure 8:**
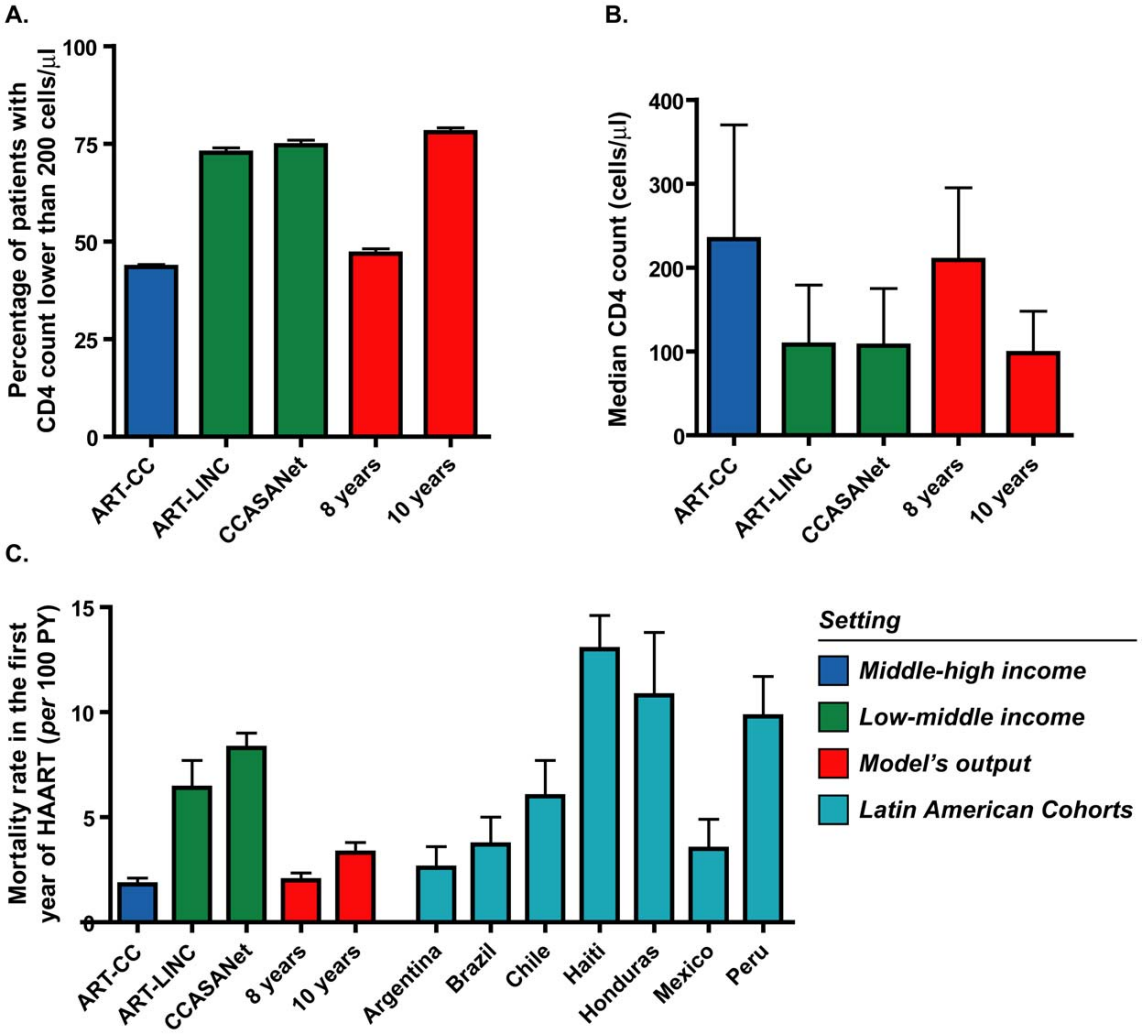
Comparison of model’s output with observations from different settings. The model was run under a fixed time to diagnosis of 8 years and 10 years. Predictions obtained using a 8-years delay resemble middle-high income settings while those obtained using 10-years delay resemble low-middle income settings for the proportion of patients with CD4 counts lower than 200 cells/µl (**A**) and median CD4 count at initiation of HAART (**B**). Predictions about mortality rate during the first year of HAART are consistent with middle-high income settings but distant from those observed in low-middle income settings (**C**). Latin American cohorts in panel C are those part of CCASAnet cohort.

The present model demonstrates a significant accuracy in simulating the natural history and treatment outcome of HIV infection for settings similar to those studied in the CASCADE Cohort and in the population-based Danish HIV Cohort Study (DHCS), against which the model was validated. Interestingly, as shown in Figure 8, when the model is set at a fixed diagnosis delay of 8 years, the modeĺs outputs closely resemble characteristics of patients observed in high income settings (ART-CC cohort [12]); while setting the model at a fixed diagnosis delay of 10 years drives outputs to resemble more closely those observed in low income settings (ART-LINC [12], CCASAnet in Latin America [32]). This suggests that the diagnosis delay by itself can play a central role in determining the epidemiological features observed in different settings.

However, while mortality rate during the first year of HAART is also accurately estimated for high-income settings, it is underestimated for low income settings in our model. As shown in Figure 8C, being set at a diagnosis delay of 10-years the model predicts a 3.3% (IC: 2.8–3,8) mortality rate in the first year of HAART that is significantly different from previous reports from these settings, which are near to 10%: for ART-LINC it was reported 6.4% (5.1–7.7) [12], 13% for Haiti [13], 8.3% (7.6–9.1) for South America [32], 8.9% (8.4–9.5) for South Africa and Malawi [33], and an average 10% in different studies from Africa as reviewed by Lawn *et al* [34]. However, this rate is highly variable even among similar settings as shown by its variability within Latin America previously reported by Tuboi *et al* [32] (Figure 8C) and might be highly dependent on variations in background and AIDS-related mortality rates as well as on disease progression rate or prevalence of comorbidities at baseline, all characteristics shown to be different between low and high-income settings [12], [32], [35] [36]. For example, our model predicts a mortality rate for untreated population of 10.9% person-year (10.4–11.5) which derives predictions highly consistent with middle-high income settings as mentioned above, but studies from Africa have had reported rates higher than 20% [37], [38], [39]. Therefore, while model’s predictions can assign a specific diagnosis delay that explain at the same time several variables observed in middle-high income settings, this is not possible in our model for the case of low income settings. As explained above we relate this fact mainly to differences in mortality rates between those settings and it also highlights that predictions obtained from our model fit more adequately to middle-high income settings.

Taking into account these limitations, our predictions still demonstrate the impact that reducing time from infection to diagnosis can have in improving clinical outcome and the consistency with observations from middle-high income settings shows that the model can provide predictions relevant to real-world settings. In this sense, our results suggest that an eight year delay stands as an inflection point between two different settings: When diagnosis delay is higher than 8 years, overall mortality rates starts to increase, life expectancy starts to decrease, capacity of recognizing HIV infection through symptomatology becomes critical, and benefits derived from not deferring treatment initiation to lower CD4 counts are lost. In contrast, achieving diagnosis before 8 years post-infection minimizes mortality rates to stable levels, maximizes life expectancy and allows prevention of early mortality through initiation of treatment at higher CD4 counts.

Importantly, our results suggest that ARV-treatment can recover life expectancy almost to the maximum achievable levels even when diagnosis delay is extremely long, indirectly demonstrating the central role that linkage to care after diagnosis plays in managing HIV infection since, although in our model every diagnosed patient who meets criteria for treatment initiation has immediate access to therapy, the effect of a delayed linkage to treatment can be estimated by moving forward to analytical settings with larger diagnosis delays. In this sense, a setting with 8 years delay for diagnosis and 1 year delay for linkage-to-care is equivalent to a 9 years diagnosis delay in terms of patient prognosis. We can observe from our estimation of patients’ age at death that even in analytical settings with the larger diagnosis delays, patients who are diagnosed and linked-to-treatment have similar life expectancy as those in analytical settings with the shortest diagnosis delays. Nevertheless, as mentioned above, in the former settings early mortality is significantly higher than in the last. Therefore, in order to prevent early mortality associated with HIV infection, achieving diagnoses within the first 8 years of infection should stand as the first goal.

It is important to note that the present model is based on individual disease progression, and as such it does not take into account transmission. Therefore, benefits derived from preventing new infections through treatment or behavioral-changes associated with knowledge of serological status are not modeled here. In this sense, it is important to highlight that additional population-level benefits can be expected from reducing diagnosis delays besides the ones predicted in the present study.

Finally, we analyzed the relationship between time to diagnosis and testing rate in order to define specific goals for achieving significant reductions in diagnosis delay. This analysis represents a key component of the study since it connects the unrealistic route of diagnosis where fixed times to diagnosis are applied, with the more realistic situation of studying variations in testing rates and relaxing the time from infection to diagnosis. As mentioned above, the set of predictions obtained under a fixed diagnosis delay are those expected when the 75% of HIV-positive individuals are diagnosed before reaching that time point of infection. In particular, according to our prediction, if an annual testing rate of 20% is reached, then 90% of infected individuals will achieve diagnosis within the first 8 years of infection. Although this testing rate is highly challenging, it may not be unfeasible if we consider the following analysis: Previous studies have estimated that the percentage of patients achieving a late diagnosis (defined as proportion of newly diagnosed individuals with CD4-count lower than 200 cells/µl) stands close to 30% [27]. Because our model estimates a time of infection of at least 8 years for patients with CD4-count lower than 200 cells/µl (Figure S3), we can infer that a 30% of late presenters corresponds with a testing coverage that allows detection of 70% of patients before 8 years of infection; which, according to the model, corresponds to a testing rate close to 10% (Figure 7). Starting from this baseline annual rate of 10%, to reach a testing rate of 20% that will allow to achieve diagnosis of 90% of HIV positive population before the first 8 years of infection may not be an impossible task if we work strongly on missed opportunities for diagnosis [9], [40], [41], [42], socio-economic status-related disparities in access to health care, social stigma and under-perception of risk that discourage people from seeking testing [16], [40], [43], [44], [45], [46], [47], [48], [49], [50], as well as on implementing more efficient testing strategies [51], [52], [53], [54], [55]. The many demonstrated benefits for the patients, previously reported and also suggested here, largely justify the effort.

## Supporting Information

Figure S1 **Viral Load-dependent CD4 count decay.** The linear function adjusted to model the reduction in CD4 count determined by viral load is shown.
(TIF)

Figure S2 **Curves describing rates of disease progression and death according to variations in viral load, CD4 count and patient’s age.** Four different scenarios are shown according to the patient’s age at the moment of infection. Although this is a continuous variable, in order to simplify the explanation only four different ages at infection are shown. For each situation, variations due to CD4 count, viral load and patient’s current age are shown. The probability of death can be obtained by multiplying the probability of disease progression by 0.45, 0.58, 0.72 or 0.85 for 25, 35, 45 and 55 years-old at infection (see Text S1).
(TIF)

Figure S3 **Median CD4 count at initiation of HAART according to time from infection to diagnosis.** Simulations were run under the following combinations of CD4 count threshold to initiate HAART and annual rate of detection through symptomatology (DTS) for the following conditions: Initiation of HAART at 200 (grey line), 350 (light blue line) and 500 (dark blue line) cells/µl; and each of them for both annual rate of DTS of 35% and 75%.
(TIF)

Figure S4 **Proportion of HIV positive individuals unaware of their infection observed at any time in a steady state situation for each of the analytical settings.** Simulations output from the same model runs analyzed for Figure 3, were also analyzed to determine the proportion of HIV-positive individuals unaware of their serological status as those who did not achieve a diagnosis out of the total of individuals living with HIV at the end of the simulation. The proportion determined for a specific analytical setting is stable across the simulation. As expected, simulations runs that differ only in the CD4 count threshold to initiate HAART give identical predictions as different treatment initiation algorithms cannot impact diagnosis of infection.
(TIF)

Figure S5 **Comparison of modeĺs output run under diagnosis route 1 (fixed diagnosis delay) and diagnosis route 3 (fixed testing rate).** For each annual testing rate, results from using the rate or using the diagnosis delay fixed at the third quartile (Q75) of the distribution of diagnosis delays are shown. Comparisons performed for mortality during the first year of HAART (upper panel), clinical stage at diagnosis and proportion of patients unaware of their infection (mid panel), and age at death (lower panel) are shown.
(TIF)

Figure S6 **Baseline distributions of variables relevant for the analysis of modeĺs predictions about the natural history of HIV infection.** The distribution of patient´s age at infection, CD4 count and viral load are shown.
(TIF)

Figure S7 **Patient´s age at progression to AIDS and death predicted in by the model in the absence of treatment.**
(TIF)

Figure S8 **Distribution of the total times to AIDS and times to death predicted by the model in the absence of treatment.**
(TIF)

Figure S9 **Times to AIDS and times to death according to patient´s age at infection death predicted by the model in the absence of treatment.**
(TIF)

Figure S10 **Baseline distributions for Mortality rates analysis.** Distribution of patientś age at infection, patientś age at initiation of HAART, viral load and CD4 count at the moment of infection are shown.
(TIF)

Figure S11 **Patientś age at death.** Distribution of patientś age at death during the simulations runs to determine the general mortality rates of HIV-positive individuals after diagnosis.
(TIF)

Figure S12 **Survival curves obtained by Kaplan Meier analysis and their natural logarithmic transformation.** Curves for the total population and separating HIV related death from other causes of death predicted by the model for HIV-positive individuals after diagnosis.
(TIF)

Figure S13 **Curve fitted to the natural logarithmic transformation of the survival curve related to AIDS mortality.** Three different functions were adjusted to each of the three age intervals shown in the figure. Functions details are provided in Text S1.
(TIF)

Text S1 (DOC)
