# Supplementary figures and images for: Estimation of HIV-Testing Rates to Maximize Early Diagnosis-Derived Benefits at the Individual and Population Level
Source: PLoS One. 2013 Jan 7;8(1):e53193. doi: 10.1371/journal.pone.0053193 (PMC3538781; doi:10.1371/journal.pone.0053193)

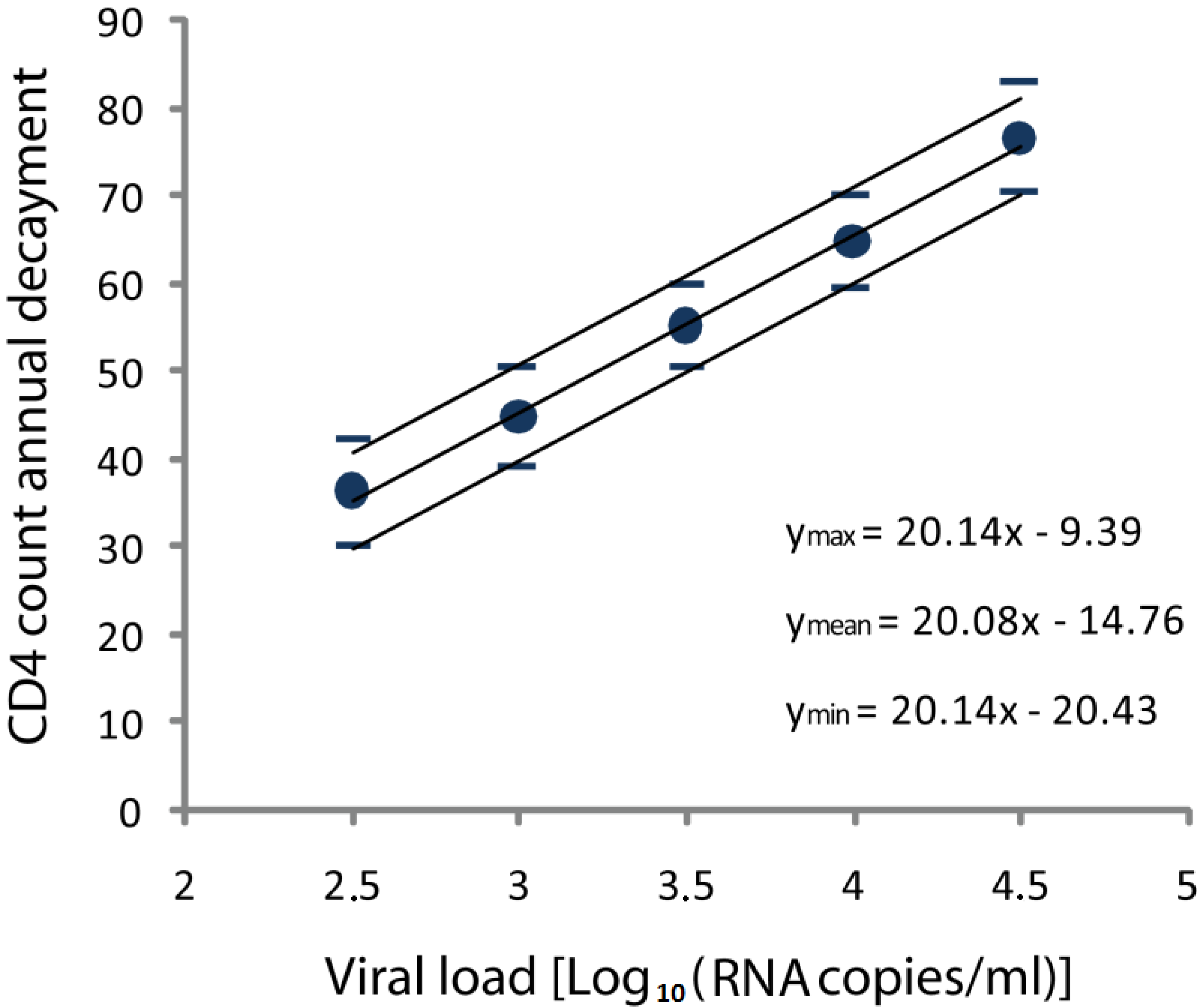

Supplement: Figure S1 — Viral Load-dependent CD4 count decay. The linear function adjusted to model the reduction in CD4 count determined by viral load is shown. (TIF) [file pone.0053193.s001.tif]

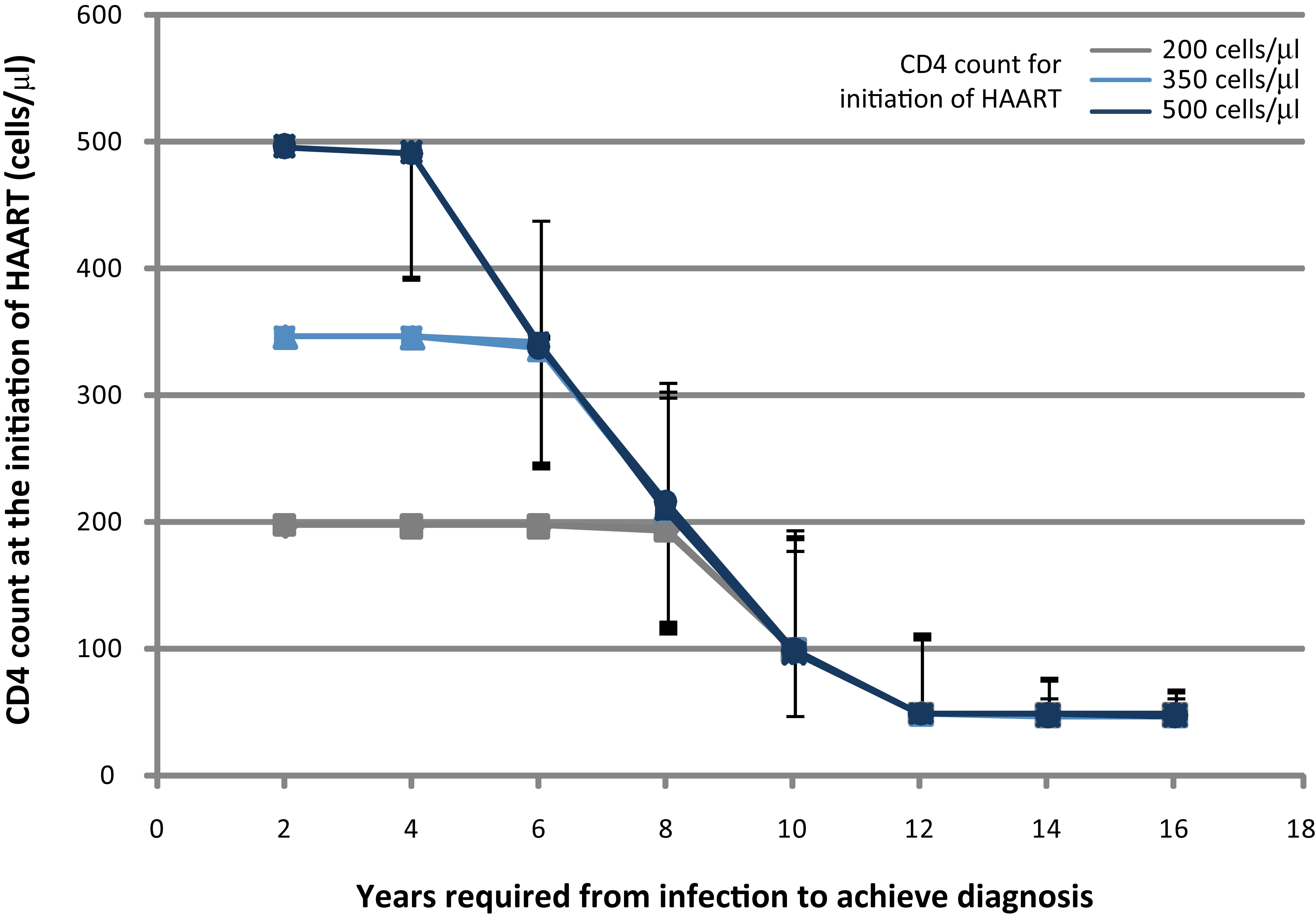

Supplement: Figure S3 — Median CD4 count at initiation of HAART according to time from infection to diagnosis. Simulations were run under the following combinations of CD4 count threshold to initiate HAART and annual rate of detection through symptomatology (DTS) for the following conditions: Initiation of HAART at 200 (grey line), 350 (light blue line) and 500 (dark blue line) cells/µl; and each of them for both annual rate of DTS of 35% and 75%. (TIF) [file pone.0053193.s003.tif]

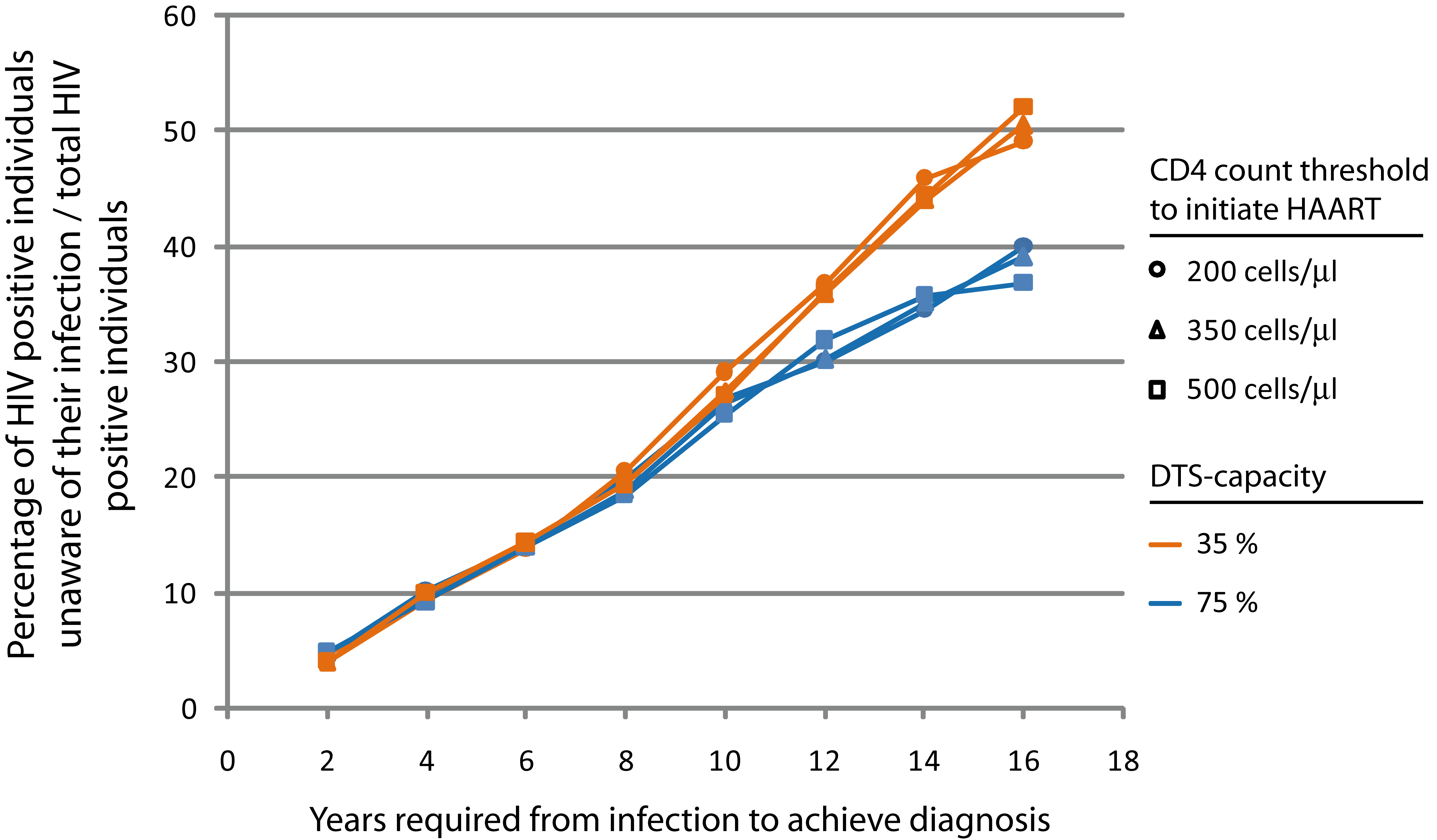

Supplement: Figure S4 — Proportion of HIV positive individuals unaware of their infection observed at any time in a steady state situation for each of the analytical settings. Simulations output from the same model runs analyzed for Figure 3, were also analyzed to determine the proportion of HIV-positive individuals unaware of their serological status as those who did not achieve a diagnosis out of the total of individuals living with HIV at the end of the simulation. The proportion determined for a specific analytical setting is stable across the simulation. As expected, simulations runs that differ only in the CD4 count threshold to initiate HAART give identical predictions as different treatment initiation algorithms cannot impact diagnosis of infection. (TIF) [file pone.0053193.s004.tif]

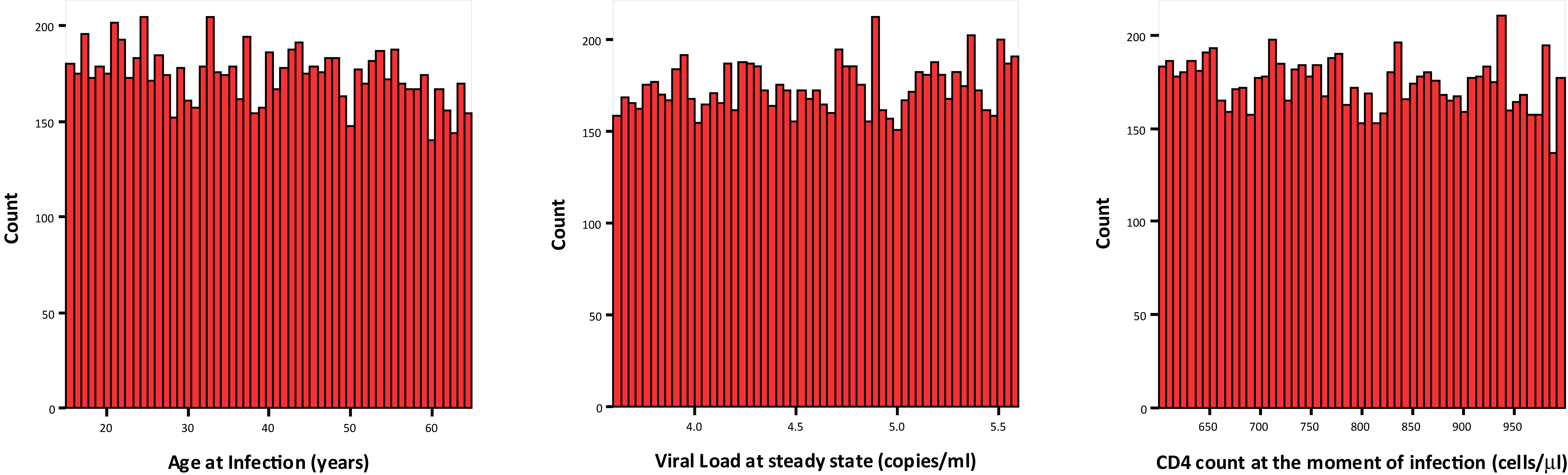

Supplement: Figure S6 — Baseline distributions of variables relevant for the analysis of modeĺs predictions about the natural history of HIV infection. The distribution of patient´s age at infection, CD4 count and viral load are shown. (TIF) [file pone.0053193.s006.tif]

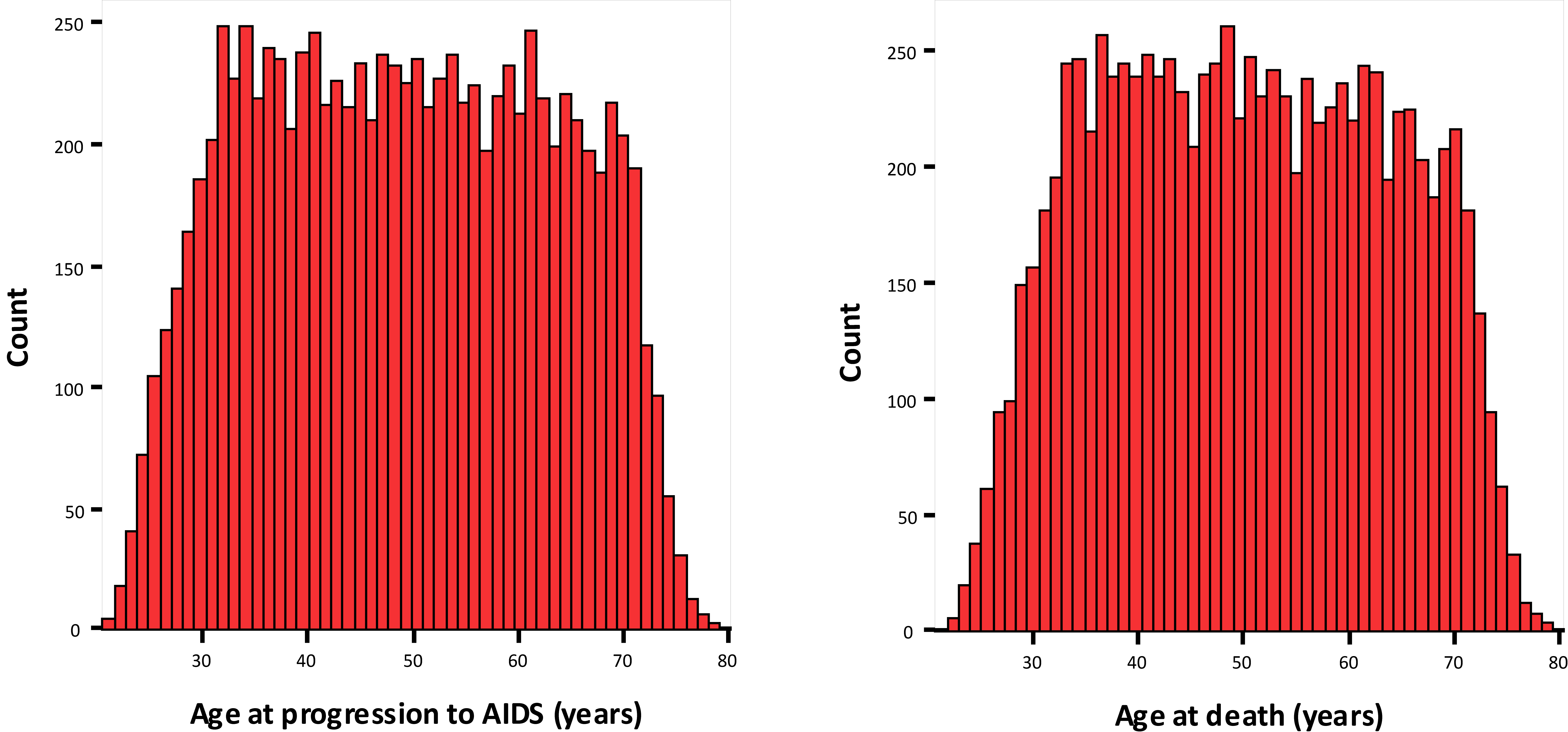

Supplement: Figure S7 — Patient´s age at progression to AIDS and death predicted in by the model in the absence of treatment. (TIF) [file pone.0053193.s007.tif]

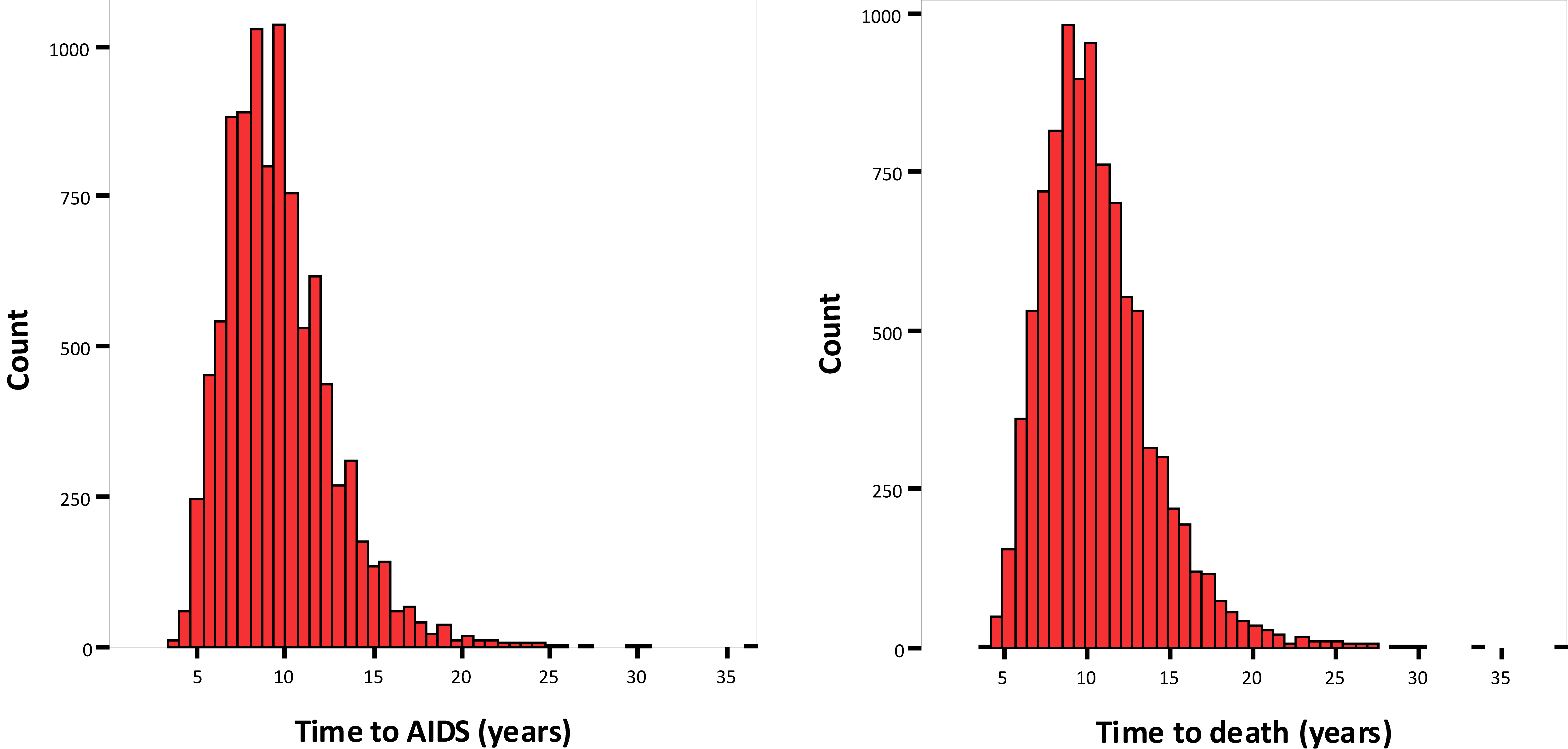

Supplement: Figure S8 — Distribution of the total times to AIDS and times to death predicted by the model in the absence of treatment. (TIF) [file pone.0053193.s008.tif]

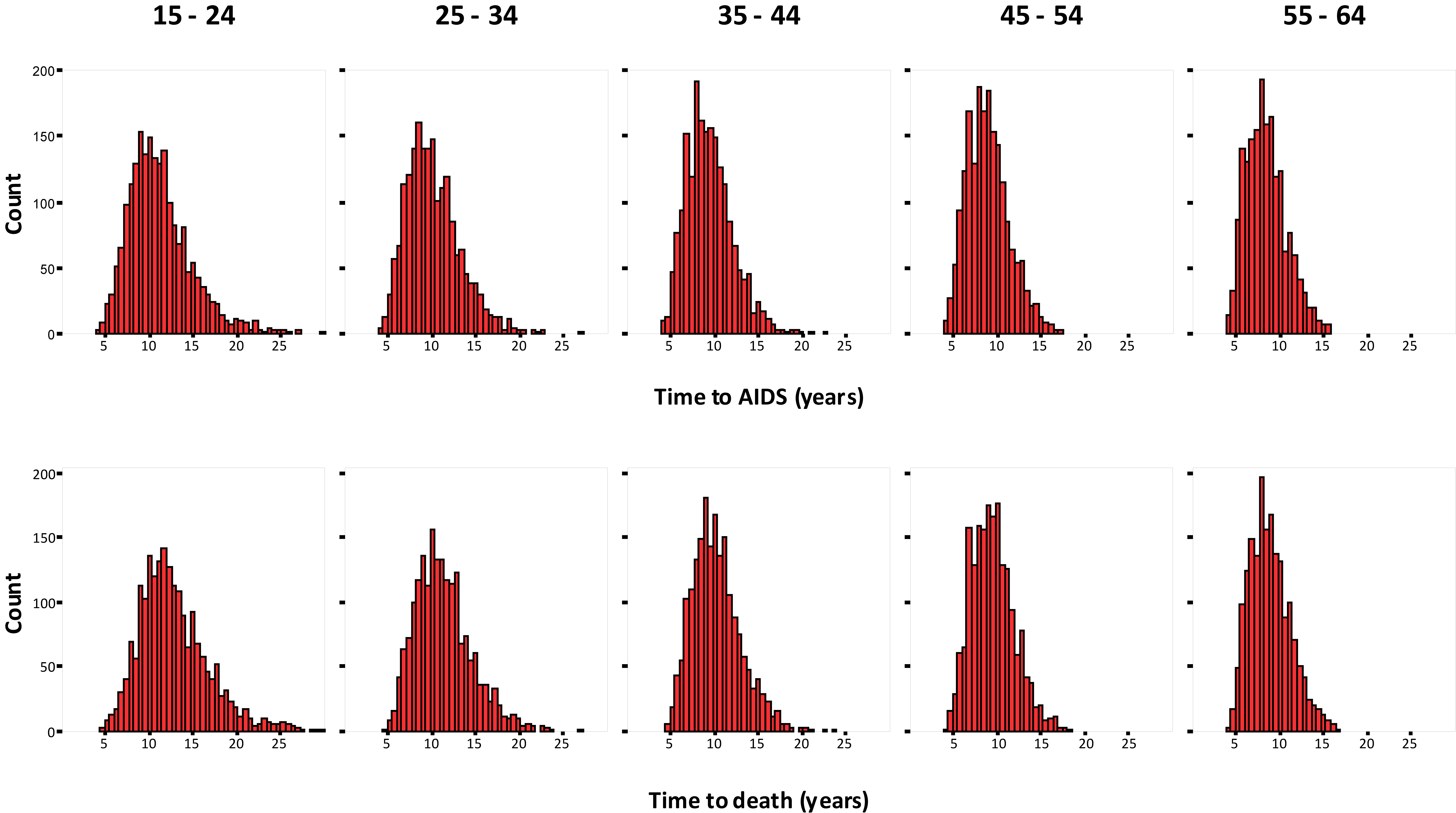

Supplement: Figure S9 — Times to AIDS and times to death according to patient´s age at infection death predicted by the model in the absence of treatment. (TIF) [file pone.0053193.s009.tif]

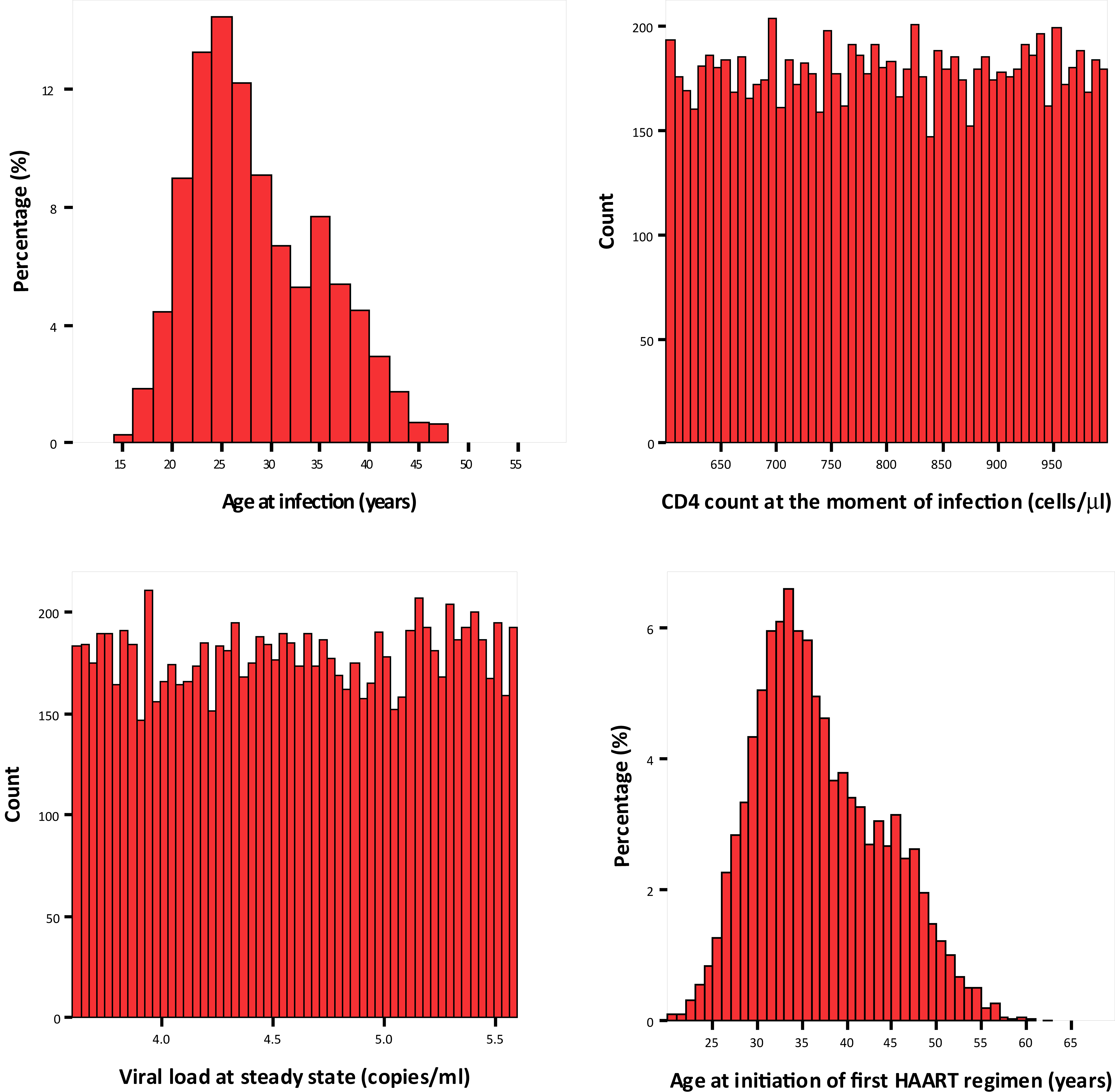

Supplement: Figure S10 — Baseline distributions for Mortality rates analysis. Distribution of patientś age at infection, patientś age at initiation of HAART, viral load and CD4 count at the moment of infection are shown. (TIF) [file pone.0053193.s010.tif]

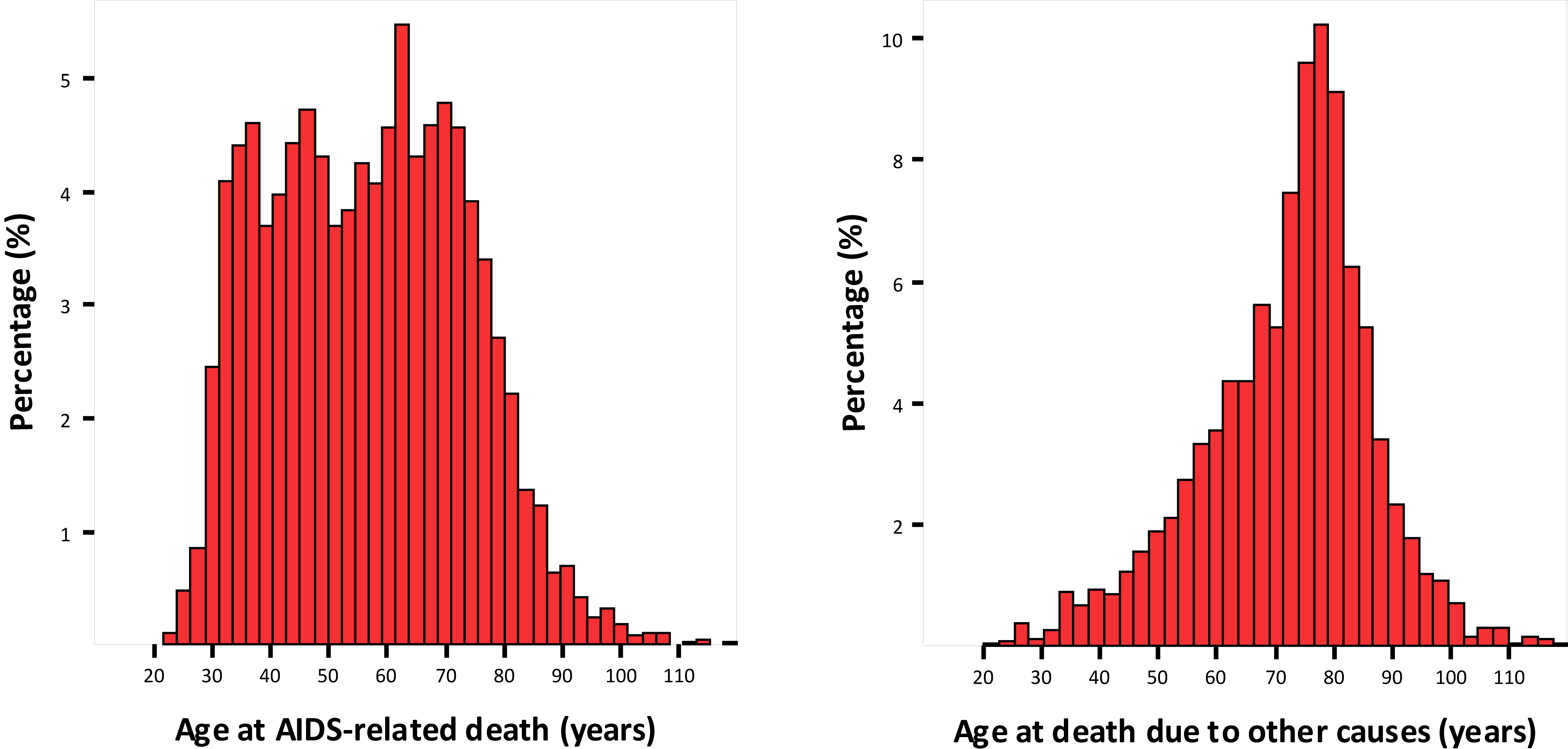

Supplement: Figure S11 — Patientś age at death. Distribution of patientś age at death during the simulations runs to determine the general mortality rates of HIV-positive individuals after diagnosis. (TIF) [file pone.0053193.s011.tif]

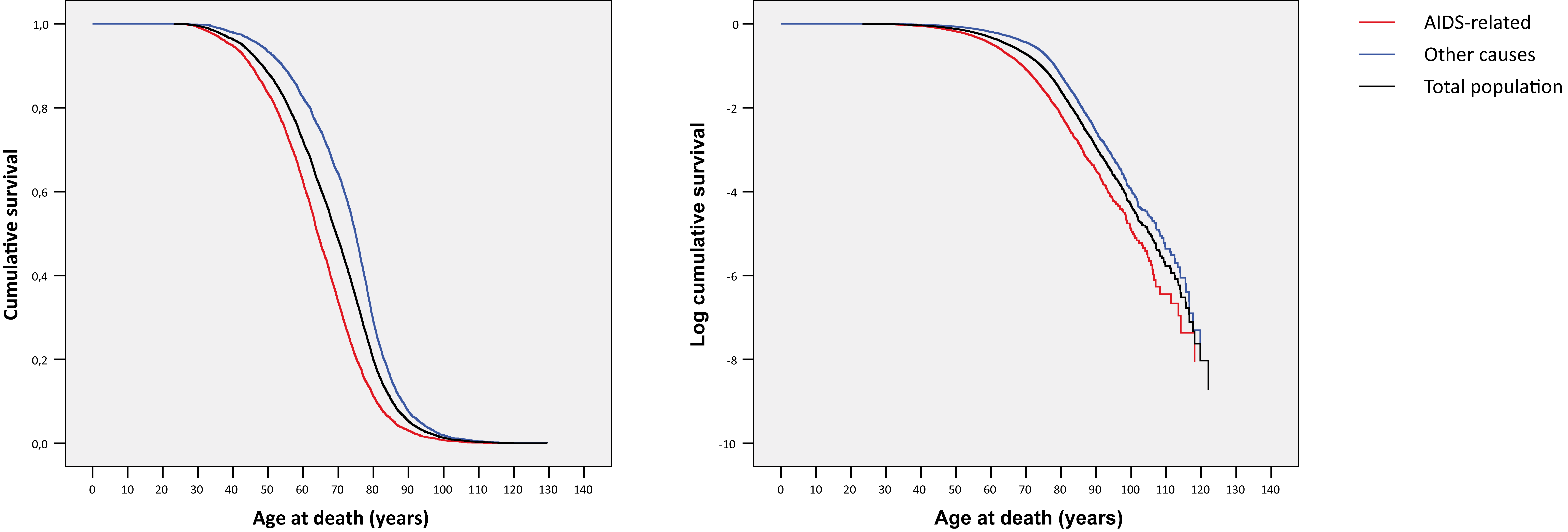

Supplement: Figure S12 — Survival curves obtained by Kaplan Meier analysis and their natural logarithmic transformation. Curves for the total population and separating HIV related death from other causes of death predicted by the model for HIV-positive individuals after diagnosis. (TIF) [file pone.0053193.s012.tif]

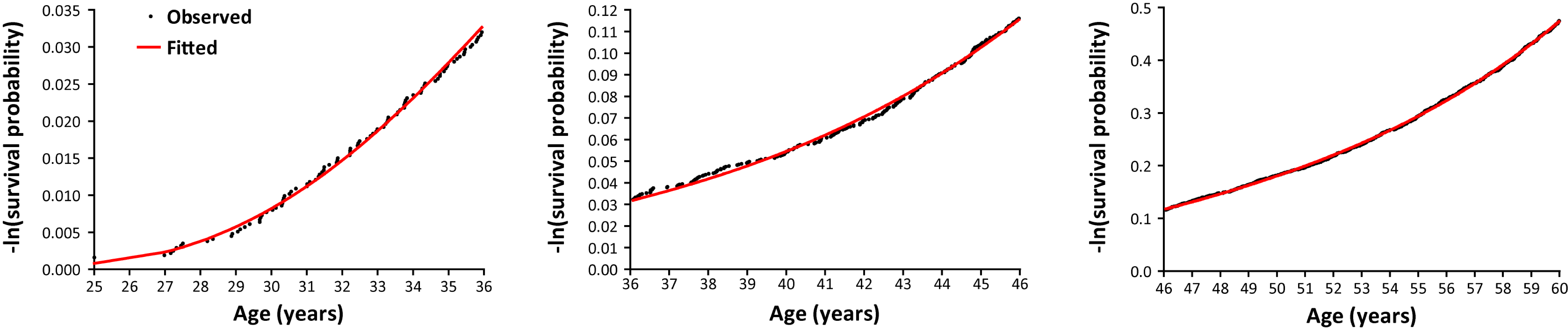

Supplement: Figure S13 — Curve fitted to the natural logarithmic transformation of the survival curve related to AIDS mortality. Three different functions were adjusted to each of the three age intervals shown in the figure. Functions details are provided in Text S1. (TIF) [file pone.0053193.s013.tif]
