# Supplementary material for: Estimation of HIV-Testing Rates to Maximize Early Diagnosis-Derived Benefits at the Individual and Population Level
Source: PLoS One. 2013 Jan 7;8(1):e53193. doi: 10.1371/journal.pone.0053193 (PMC3538781; doi:10.1371/journal.pone.0053193)
Supplement: Text S1 — (DOC) [file pone.0053193.s014.doc]

**TEXT S1.**

[I. DETAILS ON THE CONSTRUCTION OF THE MODEL 2](#_Toc339818238)

[i. Sources of data 2](#_Toc339818239)

[ii. Construction of probability functions 3](#_Toc339818240)

[VL-dependent decay of CD4 count. 3](#_Toc339818241)

[Probability of AIDS progression and death after AIDS. 4](#_Toc339818242)

[Obtaining probability of AIDS-related death. 5](#_Toc339818243)

[Probability of detection through symptomatology. 6](#_Toc339818244)

[II. DETAILS ON ASSESSMENT OF EXTERNAL CONSISTENCY 7](#_Toc339818245)

[i. Comparison of the time to AIDS and the time to death after infection predicted by the model with those reported by the Collaborative Group on AIDS Incubation and HIV Survival, Lancet (2000) Vol.1; 355(9210):1131-7. 7](#_Toc339818246)

[ii. Comparison of annual mortality rate after 25 year old predicted by the model with those reported by Lohse et al, Ann Intern Med. 2007;146:87-95. 8](#_Toc339818247)

# I. DETAILS ON THE CONSTRUCTION OF THE MODEL

## Sources of data

Age at infection was modeled according to the distribution described by Rosenberg et al [1].

The CD4 count distribution for the uninfected population was obtained from a previous study on the general population in Argentina where a median of 801 CD4 (q25:648; q75:980) was described.

The viral load at a steady state was set according to data obtained from different cohorts in Argentina and other regions: in a study performed in Buenos Aires between March 2003 and November 2005 over 321 newly diagnosed individuals, a mean viral load of 4.597 Log_10_[copies/ml] was observed (SD= 0.83021) [2]. At Hospital Fernández in Buenos Aires, a mean value of 4.021 (SD= 1.581) was observed[3] also in newly-diagnosed individuals. In a recent study performed including seven cohorts from Latin America and the Caribbean, a mean viral load of 5.0 (4.6 – 5.4) Log_10_[copies/ml] at initiation of first HAART was observed[4]. For the MACS cohort, a mean and relatively stable viral load of 4.1 Log_10_[copies/ml] was reported during the first three years after seroconvertion[5]. In the present study a value of 4.6 (3.6-5.6) Log_10_[copies/ml] was used and considered constant until initiation of HAART.

In regards to the response to HAART, variations in CD4 count and viral load as well as the frequency of discordant responses were modeled according to rates described previously for the ART-LINC cohort [6]. This is a cohort of patients from low-income settings with a median age of 35 years, median baseline CD4 cell count of 137 cells per microliter and viral load of 5.1 Log_10_[copies/ml] at initiation of HAART.

The recovering of the CD4 count after successful HAART was modeled according to Mocroft et al [7]. The model was developed in order to allow an increment in 60 CD4 cells per year as observed in the EUROSIDA cohort with the restriction that the CD4 count cannot exceed the value corresponding to the uninfected state.

After therapy failure, the viral load at rebound was modeled according to Le Moing *et al* [8] and Deeks *et al* [9]. Both studies analyzed virological rebound after failure to protease inhibitor-containing regimens.

Toxicity and rebound rates, as well as increased risk of disease progression related to past AIDS, were those observed by the EUROSIDA cohort [10].

Suppression rate was estimated according to CCASAnet cohort [4,11], PUMA cohort[12] and ART-LINC [6]. The observed rates were consistent in the different cohorts with around 50% of individuals achieving undetectable viral load, and 25% showing viral load reduction to levels between 50 and 400 RNAcopies/ml.

## Construction of probability functions

VL-dependent decay of CD4 count. We first applied the mathematical function described by Cook *et al* [13] and later the one by Sanders *et al* [14]. However, this function takes a value of zero when viral load equals 2.36 Log_10_[copies/ml] and therefore the CD4 count variation after viral load drops below that limit becomes increasingly positive. To correct this, we applied a mathematical function fitted to the data described by Mellors *et al* [15] where the annual decay rate was estimated for different ranges of viral load (Supplementary Figure S1). Therefore, the monthly rate of CD4 decay according to viral load is determined by:

;


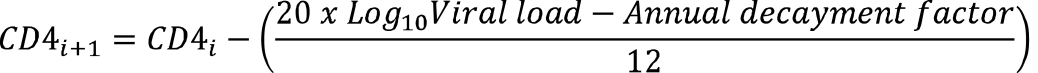


where the *Annual decay factor* is a mean value of 14.76 (95%CI=9.39-20.43). This function has the same limitation as the one described by Cook *et al* [13] but the viral load at which the CD4 net variation becomes positive is 0.7 log (0.5-1.0) which is below the limit of detection applied in our model (50 copies/ml = 1.7 log).

Probability of AIDS progression and death after AIDS. The mathematical function applied for modeling the probability of AIDS progression was based on the one reported by Phillips *et al* [16] while the one applied for the probability of death after AIDS was based on a report by Schneider *et al* [17]. Both functions were modified in order to consider variations in risk associated to different age at seroconvertion reported for the CASCADE cohort [18].

The general probability for transition between states is defined as


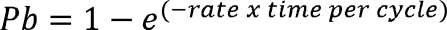


Considering that every cycle of the simulation represents a time step of one month, and that rates may be affected by a modified risk factor (Z), then


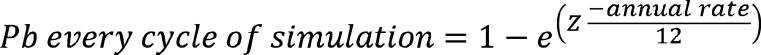


The annual rate of progression to AIDS described by Phillips et al [16] for the CASCADE cohort is given by the function:


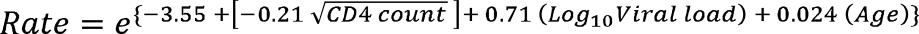


Based on the observation reported also for CASCADE [18], where progression was increased by a factor of 1.32 every 10-years increase in the patient´s age at infection, and considering the minimal patient´s age at infection at 15 year-old, we obtained a function that determines the increased risk of progression (Z_AIDS_) according to the next function:


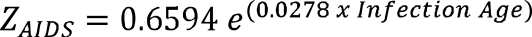


Of note, when age at infection equals 15, then Z_AIDS_ equals 1 and increase by a factor of 1.32 every 10 years. In the case of the probability function for death after AIDS, Schneider *et al* [17] reported that 25% of individuals die after AIDS diagnosis in 0.56 years in the no-treatment/monotherapy era, which is the closer setting to the natural infection. That percentage corresponds to a monthly rate of 0.0428, which is independent of any other parameter. Then, we included an increased risk of 1.47 folds every 10-years increase in age at infection[18]. Considering again the minimal age at infection at 15 year-old, the Z_Death_ is defined as:


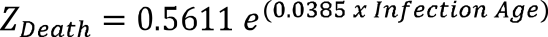


This rate gives a mean survival time after development of AIDS of 1.17 years.

Obtaining probability of AIDS-related death. The function that determines the probability of disease progression (P_AIDS_) and death after AIDS (P_Death/AIDS_) according to CD4 count, viral load, current patient’s age and patient’s age at infection were provided above. The probability of AIDS-related death can be obtained simply by multiplying both probabilities since:

P_(Death/AIDS)_ = (P_AIDS_ ∩ P_Death_)/P_(AIDS)_ & P_(AIDS/Death)_ = (P_AIDS_ ∩ P_Death_)/P_(Death)_

And therefore: P_(Death/AIDS)_ = P_(AIDS/Death)_ P_(Death)_ / P_(AIDS)_  ⇒ P_(Death)_ = P_(Death/AIDS)_ P_(AIDS)_/ P_(AIDS/Death)_

Since: P_(AIDS/Death)_=1, then P_(Death)_ = P_(Death/AIDS)_ P_(AIDS)_ , as mentioned above

See Supplementary Figure S2 for a detailed analysis of how rates of disease progression and death change according to variations in viral load, CD4 count and patient’s age.

Probability of detection through symptomatology. This probability was defined as a linear function dependent on CD4 count, based on the one previously described by Sanders *et al* [14], but modified in order to allow a maximum rate of detection of 35% or 75% (DTS efficiency) at CD4 count equal or lower to 50 cells/µl and a minimum of 0% at CD4 count equal or higher than 350 cells/µl.

# II. DETAILS ON ASSESSMENT OF EXTERNAL CONSISTENCY

## Comparison of the time to AIDS and the time to death after infection predicted by the model with those reported by the Collaborative Group on AIDS Incubation and HIV Survival, Lancet (2000) Vol.1; 355(9210):1131-7.

In order to perform this analysis, we set up the model with the following parameters:

***Age at infection:*** *U(15; 64).*

***Viral load at steady state:*** *U(3.6; 5.6).*

***CD4 count at the moment of infection:*** *U(600; 1000).*

***Age at diagnosis (A_dx_):*** Defined as A_dx_ = A_inf_ + 400, with A_inf_: age at infection, in order to prevent diagnosis and to analyze natural history of infection.

***Probability of being diagnosed through symptomatoloy*** = 0

***Simulations:*** 10,000 monte-carlo simulations with 1,800 cycles per run

**Output analysis**

In order to describe the population under analysis, we show the distribution of values sampled by the model at the beginning of the simulations for variables defining patients´ age at infection, viral load at steady state and CD4 count at the moment of infection in supplementary figure S6. As expected, the distributions reflect the initial set up of the model for those parameters described above. We also show the posterior distributions obtained for patient´s age at progression to AIDS and death in supplementary figure S7 for the total population under analysis. Using the model´s output, time to AIDS and time to death were calculated and analyzed for the total population as well as classifying patients according to age at infection. Finally, time to AIDS and time to death according to patient´s age at infection were compared with those previously reported for the CASCADE Cohort [18]. The distribution of the total times to AIDS and times to death are shown in supplementary figure S8 while times to AIDS and times to death according to patient´s age at infection are shown in supplementary figure S9. As shown in figure 2 of the main manuscript, our predictions are highly accurate, following the trend observed in CASCADE cohort although tend to overestimate the time to AIDS and death for ages at infection higher than 35.

ii. Comparison of annual mortality rate after 25 year old predicted by the model with those reported by Lohse et al, Ann Intern Med. 2007;146:87-95.

Lohse *et al.* [19] estimated the absolute mortality rate (mortality rate for the general population subtracted from the mortality rate for patients with HIV infection) after diagnosis and during three different eras of HAART. In the present analysis we compare model´s prediction with those reported for patients treated between 2000 and 2005. In order to perform this analysis, we set up the model with the following parameters:

***Age at infection:*** (18 to 56), Median=26.

***Viral load at steady state:*** *U(3.6; 5.6).*

***CD4 count at the moment of infection:*** *U(600; 1000).*

***Annual testing rate:*** 10%

***Treatment CD4 threshold:*** 200 cells/ul

***Simulations:*** 10,000 monte-carlo simulations with 1,800 cycles per run

**Output analysis**

Anna faris Kathleen Robertson. In order to describe the population under analysis, we show the distribution of values sampled by the model at the beginning of the simulations for variables defining patients´ age at infection, viral load at steady state and CD4 count at the moment of infection in supplementary figure S10. These distributions reflect the initial set up of the model for those parameters described above. In addition, distribution of patients’ age at the initiation of the first HAART regiment is shown in supplementary figure S10. Also, posterior distributions obtained for patients´ age at death are shown in supplementary figure S11, separating AIDS-related causes of death from those not related to AIDS.

Using model´s output, we obtained the survival curves for the total population, as well as for death related to AIDS and death not related to AIDS, censoring the corresponding cases in each of the last two analyses. Survival curves obtained by Kaplan Meier analysis are shown in supplementary figure S12. If mortality rate is constant across time, then the logarithm transformation of the survival function should be linearly related, with a slope equal to the annual mortality rate. The lack of linearity shown in the natural logarithm transformation of survival curves (supplementary figure S12) implies that the overall mortality rate is not constant over time. Our analysis of the natural logarithm transformed survival curve derived from the AIDS-related mortality rates show that it can be fitted to the following function:


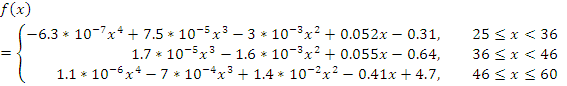


With being the patient´s age expressed in years.


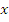

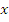


The mortality rate at each time point is the slope of the tangent line to the curve and can be obtained from the derivative of the function described above:


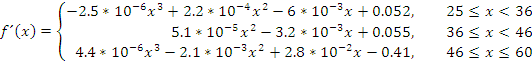


The distribution of the ln transformed AIDS-related survival probabilities obtained from the model and the adjusted function are shown in supplementary figure S13. The average mortality rate per 5-year range obtained from integring the derivative function are compared with those reported by Lohse *et al* for each five-year age interval in the figure 2 of the main manuscript.


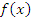

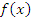

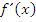

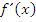


**REFERENCES**

1. Rosenberg PS (1995) Scope of the AIDS epidemic in the United States. Science 270: 1372-1375.

2. Dilernia DA, Jones L, Rodriguez S, Turk G, Rubio AE, et al. (2008) HLA-driven convergence of HIV-1 viral subtypes B and F toward the adaptation to immune responses in human populations. PLoS One 3: e3429.

3. Ameal F, Pemoff R, Cando O, Pérez H, Cahn P (2005) Diez años de testeo para HIV en el Hospital Fernández. Actualizaciones en SIDA 13: 150-156.

4. Tuboi SH, Schechter M, McGowan CC, Cesar C, Krolewiecki A, et al. (2009) Mortality during the first year of potent antiretroviral therapy in HIV-1-infected patients in 7 sites throughout Latin America and the Caribbean. J Acquir Immune Defic Syndr 51: 615-623.

5. Lyles RH, Munoz A, Yamashita TE, Bazmi H, Detels R, et al. (2000) Natural history of human immunodeficiency virus type 1 viremia after seroconversion and proximal to AIDS in a large cohort of homosexual men. Multicenter AIDS Cohort Study. J Infect Dis 181: 872-880.

6. Tuboi SH, Brinkhof MW, Egger M, Stone RA, Braitstein P, et al. (2007) Discordant responses to potent antiretroviral treatment in previously naive HIV-1-infected adults initiating treatment in resource-constrained countries: the antiretroviral therapy in low-income countries (ART-LINC) collaboration. J Acquir Immune Defic Syndr 45: 52-59.

7. Mocroft A, Phillips AN, Gatell J, Ledergerber B, Fisher M, et al. (2007) Normalisation of CD4 counts in patients with HIV-1 infection and maximum virological suppression who are taking combination antiretroviral therapy: an observational cohort study. Lancet 370: 407-413.

8. Le Moing V, Chene G, Carrieri MP, Alioum A, Brun-Vezinet F, et al. (2002) Predictors of virological rebound in HIV-1-infected patients initiating a protease inhibitor-containing regimen. Aids 16: 21-29.

9. Deeks SG, Hecht FM, Swanson M, Elbeik T, Loftus R, et al. (1999) HIV RNA and CD4 cell count response to protease inhibitor therapy in an urban AIDS clinic: response to both initial and salvage therapy. Aids 13: F35-43.

10. Mocroft A, Phillips AN, Soriano V, Rockstroh J, Blaxhult A, et al. (2005) Reasons for stopping antiretrovirals used in an initial highly active antiretroviral regimen: increased incidence of stopping due to toxicity or patient/physician choice in patients with hepatitis C coinfection. AIDS Res Hum Retroviruses 21: 527-536.

11. Socias ME, Sued O, Laufer N, Lazaro ME, Mingrone H, et al. (2011) Acute retroviral syndrome and high baseline viral load are predictors of rapid HIV progression among untreated Argentinean seroconverters. J Int AIDS Soc 14: 40.

12. Zala C, Rustad CA, Chan K, Khan NI, Beltran M, et al. (2008) Determinants of treatment access in a population-based cohort of HIV-positive men and women living in Argentina. Medscape J Med 10: 78.

13. Cook J, Dasbach E, Coplan P, Markson L, Yin D, et al. (1999) Modeling the long-term outcomes and costs of HIV antiretroviral therapy using HIV RNA levels: application to a clinical trial. AIDS Res Hum Retroviruses 15: 499-508.

14. Sanders GD, Bayoumi AM, Sundaram V, Bilir SP, Neukermans CP, et al. (2005) Cost-effectiveness of screening for HIV in the era of highly active antiretroviral therapy. N Engl J Med 352: 570-585.

15. Gupta P, Mellors J, Kingsley L, Riddler S, Singh MK, et al. (1997) High viral load in semen of human immunodeficiency virus type 1-infected men at all stages of disease and its reduction by therapy with protease and nonnucleoside reverse transcriptase inhibitors. J Virol 71: 6271-6275.

16. Kawashima Y, Pfafferott K, Frater J, Matthews P, Payne R, et al. (2009) Adaptation of HIV-1 to human leukocyte antigen class I. Nature 458: 641-645.

17. Schneider MF, Gange SJ, Williams CM, Anastos K, Greenblatt RM, et al. (2005) Patterns of the hazard of death after AIDS through the evolution of antiretroviral therapy: 1984-2004. Aids 19: 2009-2018.

18. (2000) Time from HIV-1 seroconversion to AIDS and death before widespread use of highly-active antiretroviral therapy: a collaborative re-analysis. Collaborative Group on AIDS Incubation and HIV Survival including the CASCADE EU Concerted Action. Concerted Action on SeroConversion to AIDS and Death in Europe. Lancet 355: 1131-1137.

19. Lohse N, Hansen AB, Pedersen G, Kronborg G, Gerstoft J, et al. (2007) Survival of persons with and without HIV infection in Denmark, 1995-2005. Ann Intern Med 146: 87-95.
